# Supplementary material for: Probing cytoskeletal modulation of passive and active intracellular dynamics using nanobody-functionalized quantum dots
Source: Nat Commun. 2017 Mar 21;8:14772. doi: 10.1038/ncomms14772 (PMC5364406; doi:10.1038/ncomms14772)
Supplement: Supplementary Information — Supplementary Figures, Supplementary Tables, Supplementary Methods, Supplementary Notes and Supplementary References [file ncomms14772-s1.pdf]

SUPPLEMENTARY INFORMATION

Supplementary Figure 1

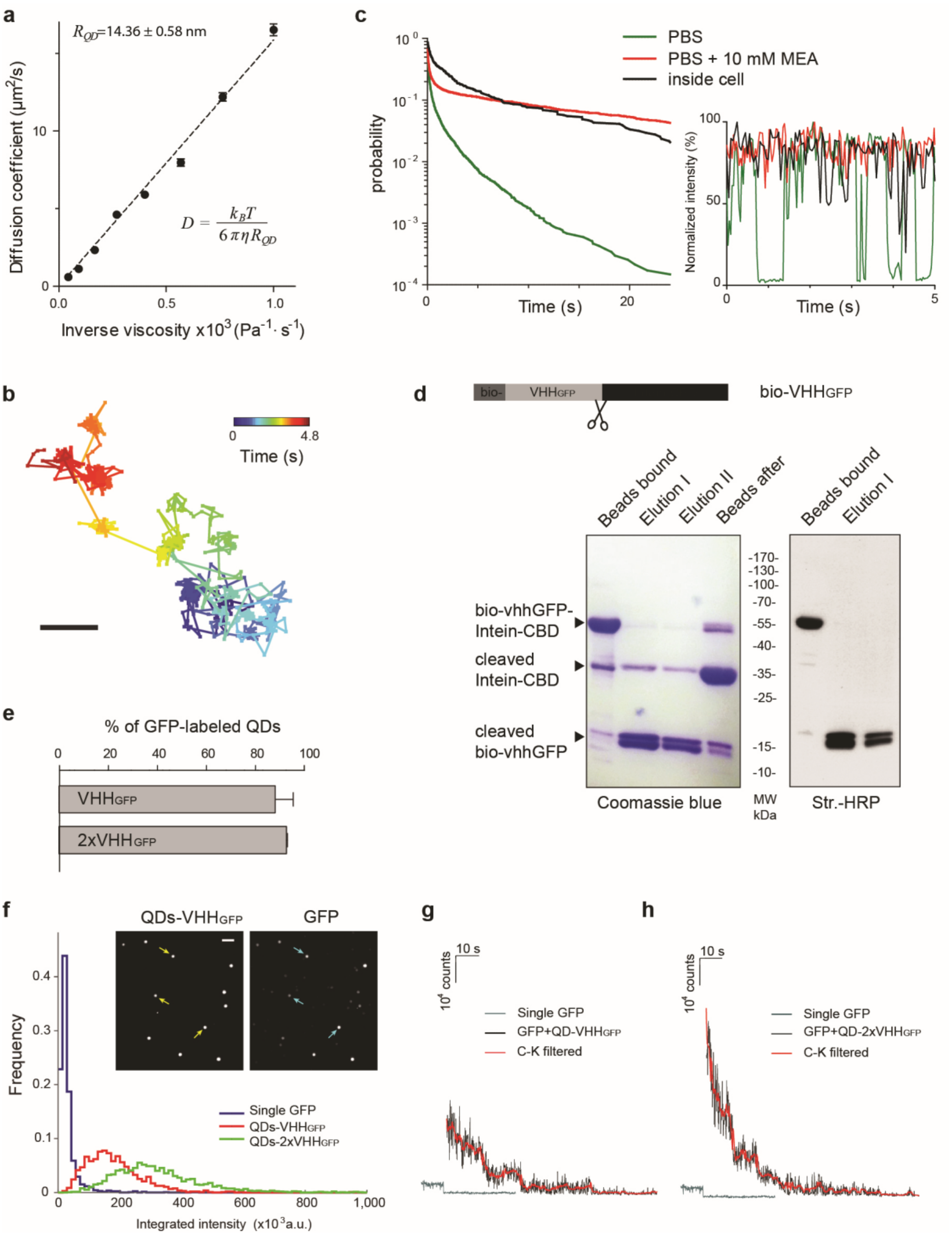

Supplementary Figure 1: Preparation and tunable loading capacity of functionalized QDs. (**a**) Diffusion coefficient of QDs measured using SPT as a function of inverse viscosity. We used aqueous glycerol solutions of different concentration to modulate viscosity and averaged MSD analysis of trajectories to derive diffusion coefficient. The value of Stokes-Einstain radius

was derived from fitting to equation presented on the plot.

(**b**) Example trajectory of non-functionalized QD inside cell showing switching between phases of slow and fast diffusion. Scale bar: 200 nm.

(**c**) Left: on-time probability distribution of QDs versus time, defined as the probability that a QD will remain "on" after indicated time interval in PBS solution (green), PBS with 10 mM MEA (red) and inside COS-7 cells attached to KIF5B (black) (N=1678, 1261 and 74 tracks). Right: example background subtracted and normalized fluorescence time traces of individual QDs in each condition.

(**d**) Top: schematic representation of bio-VHH_{GFP}-Intein-CBD fusion proteins. Cleavage of Intein-CBD purification tag is induced by incubation with DTT. Bottom: purification and elution of bio-VHH_{GFP}. Thiol-induced cleavage of the recombinant proteins (elution I and II) is verified by SDS-PAGE and by coomassie blue staining. Biotinylation is confirmed by western blot with streptavidin-HRP (str.-HRP).

(**e**) Percentage of QDs-VHH_{GFP} and QDs-2xVHH_{GFP} colocalized with GFP (n=975, n=1433 respectively). Error bars indicate SD.

(**f**) Histograms of the initial fluorescence intensities of GFP molecules bound to single VHH_{GFP} (blue), QDs-VHH_{GFP} (red) and QDs-VHH_{GFP} (2x) (green) (see Table S2 for details) Inset: representative TIRFM images of single immobilized QDs-VHH_{GFP} (left) bound to GFP (right). Scale bar: 2 μm.

(**g**,**h**) Example time trace of GFP intensity colocalized with **g** QD-VHH_{GFP} or **h** QD-2xVHH_{GFP} (black line) and Chung-Kennedy edge-preserving filtered trace (red). Stepwise photobleaching of a single GFP molecule immobilized with VHH_{GFP} is shown below (grey).

Supplementary Figure 2

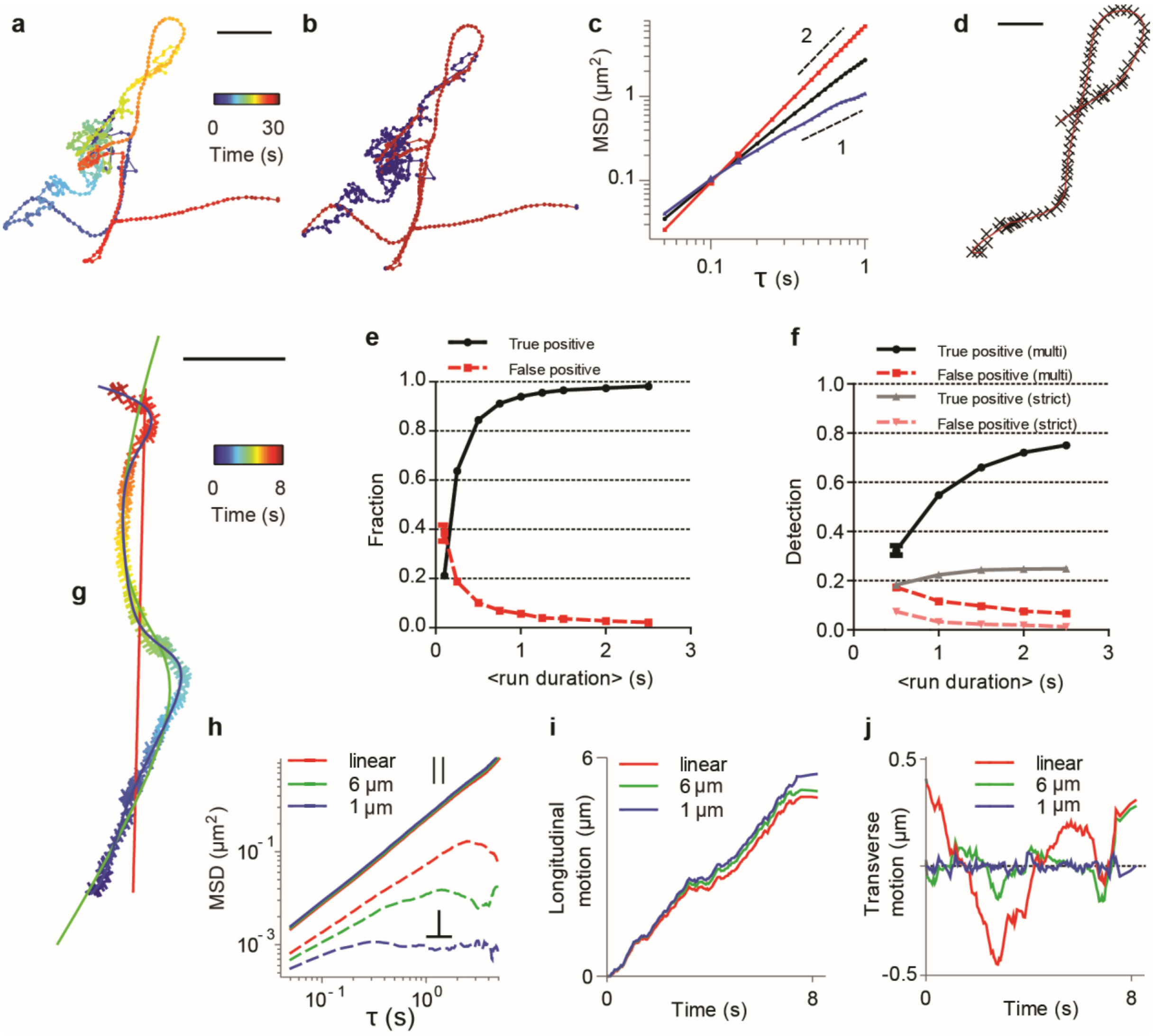

Supplementary Figure 2: Motion analysis of QDs coupled to different kinesins.

(**a**) Example trajectory of QD-VHH$_{GFP}$ coupled to Kinesin-2-GFP, color-coded for time from blue to red as indicated by the color bar (20 frames per second). Scale bar: 2 μm.

(**b**) The same trajectory split into segments of directed (red) and random (blue) motion, using the "multi-scale directional filtering" algorithm.(see methods).

(**c**) MSD of the same full trajectory (black) and its directed (red) and random (blue) motion segments. Approximate power-law slopes of 1 and 2 are indicated.

(**d**) Example of B-spline fitting (red curve) of the directed motion segment (black crosses) from **b**. Scale bar: 1 μm.

(**e**) Fractions of true (black solid line) and false positive (red dashed line) frames containing directional runs detected by "strict directional filtering" algorithm (see methods) on the artificial dataset containing directional runs interspersed with random motion intervals.

(**f**) Fractions of true (black and grey solid lines) and false positive (red and light pink dashed line) frames detected as directional runs by “multi-scale” (black and red) and “strict directional filtering” (grey and pink) algorithms (see methods) on an artificial dataset containing noisy directional runs interspersed with random motion intervals.
(**g**) Example of a directed motion trajectory of kinesin-1 (time color-coded crosses) with linear (red curve) and B-spline approximations. In the latter case the distances between internal control points are 6 μm (green) and 1 μm (dark blue). Scale bar: 1 μm.
(**h**) MSD of longitudinal (solid lines) and transverse (dashed lines) components of trajectory shown in **e** for different approximations (linear, B-spline - 6 μm, B-spline - 1 μm).
(**i-j**) Decomposition of the trajectory shown in **e** into longitudinal **g** and transverse **h** components for different approximations.

Supplementary Figure 3

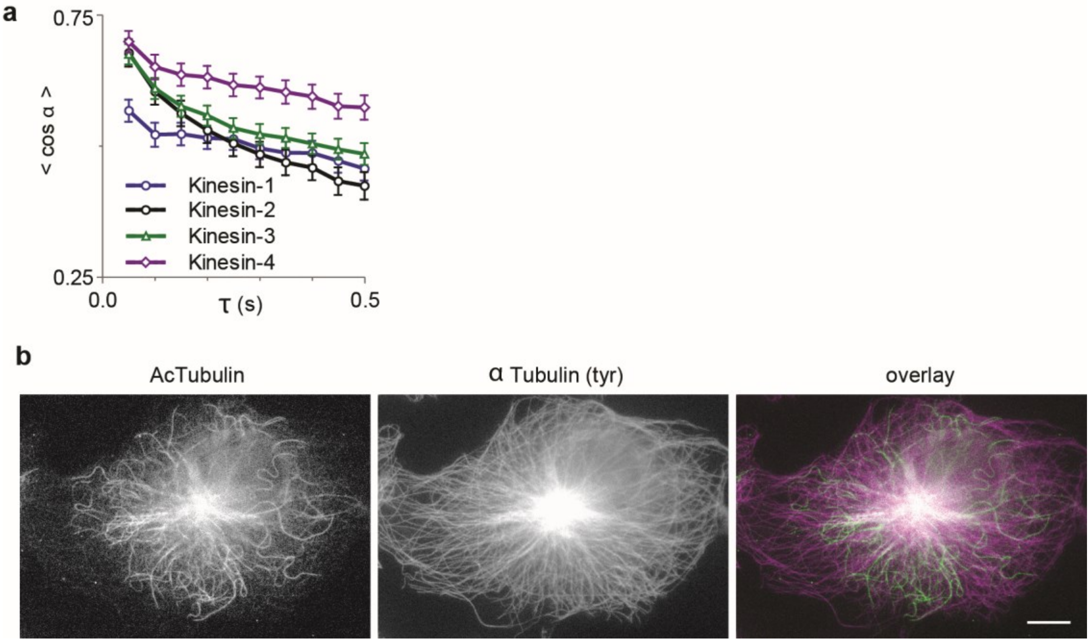

Supplementary Figure 3: Kinesin-1 prefers a subset of microtubules.

(**a**) Average direction autocorrelation (cosine between consecutive displacements) as a function of time delay (n is the same as in Fig. 3d).

(**b**) COS-7 cell fixed and stained for acetylated (left) and tyrosinated (middle) tubulin. Scale bar: 5 μm.

SUPPLEMENTARY TABLES

Supplementary Table 1: Electroporation parameters used for intracellular QD delivery

Parameter	Poring pulse	Transfer pulse
Voltage (V)	200	50
Length (ms)	5	50
Interval (ms)	50	10
Number	2	10
Decay Rate (%)	10	40

Supplementary Table 2: Fluorescent intensity values of GFP molecules attached to different probes

	single VHH_{GFP}	QDs-VHH_{GFP}	QDs-2xVHH_{GFP}
Fluorescent intensity, counts x 10^5, ± SD	0.30 ± 0.42	1.8 ± 0.9	3.2 ± 1.6
Number of spots	1541	1071	1835

Supplementary Table 3: Characterization of directed motion segments of different kinesins

	Kinesin-1 (KIF5B)	Kinesin-2 (KIF17)	Kinesin-3 (KIF1A)	Kinesin-4 (KIF21B)
Total number of tracks	91	134	181	26
Average localization, nm	3.8 ± 0.7	3.9 ± 0.4	4.6 ± 1.2	4.2 ± 0.50
strict directional filtering				
Number of segments (runs)	123	161	195	197
Speed (μm/s)	1.32 ± 0.03	2.39 ± 0.08	1.93 ± 0.05	1.16 ± 0.03
Run length (μm)	1.05 ± 0.04	2.49 ± 0.16	1.73 ± 0.07	1.21 ± 0.06
Run duration (s)	0.81 ± 0.03	1.03 ± 0.06	0.90 ± 0.03	1.04 ± 0.04
multi-scale directional filtering				
Number of segments (runs)	104	146	151	144
Speed (μm/s)	1.03 ± 0.04	1.59 ± 0.08	1.45 ± 0.06	0.98 ± 0.03
Run length (μm)	2.13 ± 0.12	3.14 ± 0.24	2.68 ± 0.15	2.19 ± 0.12
Run duration (s)	1.16 ± 0.13	1.91 ± 0.10	1.93 ± 0.09	2.39 ± 0.14
Transverse MSD scaling exponent x 10^{-2}	6.5 ± 0.5	11.2 ± 2.1	9.2 ± 0.6	8.6 ± 0.9
Longitudinal MSD scaling exponent	1.94 ± 0.02	1.95 ± 0.02	1.91 ± 0.02	1.95 ± 0.01

The data are presented for two cases. The first is “strict directional filtering” when the trajectories were filtered based on angle between two consecutive displacements. The second is

“multi-scale directional filtering”, described in Materials and Methods section. SEM is indicated.

Supplementary Table 4: Local curvature values of directed motion segments of kinesin trajectories

	Kinesin-1 (KIF5B)	Kinesin-2 (KIF17)	Kinesin-3 (KIF1A)	Kinesin-4 (KIF21B)
N	4247	8922	7965	6041
Characteristic decay of exponential fit (μm^{-1})	0.56 ± 0.03	0.18 ± 0.01	0.29 ± 0.01	0.25 ± 0.01
Median of curvature (μm^{-1})	0.49	0.36	0.37	0.34
Average of curvature (μm^{-1})	1.11 ± 0.04	1.16 ± 0.03	1.13 ± 0.03	0.95 ± 0.03

Original tracks were filtered using multi-scale directional filtering and fitted with B-spline with 1 µm distance between knots. Spline curves were re-sampled with 50 nm step and the curvature of 3 adjacent points was calculated. SEM is indicated.

SUPPLEMENTARY METHODS

cDNA constructs and antibody

For cloning of bio-VHH_{GFP} construct in pMXB10 vector, vhhGFP4 sequence[1] was amplified by PCR. Biotin acceptor sequence (bio-tag)[2] was produced by annealing of complementary oligos (forward: ATTCCATATGTCCGGCCTGAACGACATCTTCGAGGCTCAGAAAATCGAATGGCACGAAAAGCTTTCTTC; reverse: GAAGAAAGCTTTTCGTGCCATTCGATTTTCTGAGCCTCGAAGATGTCGTTCAGGCCGGACATATGGAAT). EGFP and BirA were cloned by PCR from pEGFP-C1 (Clontech) and pCI-Neo-BirA[2], respectively, and inserted into linearized pET-SUMO via TA-ligation (Champion™ pET SUMO Expression System, Invitrogen). GFP-actin in p-beta-actin vector was produced by subcloning actin cDNA from pEGFP-actin[3] into p-beta-actin-GFP (AscI/SalI). The identity of the new constructs was confirmed by sequencing. Rat KIF1A(1-383) and rat KIF21B(1-415) cDNAs were cloned into p-beta-actin-FRB-GFP with AscI/EcoRI and EcoRI/SalI respectively. KIF5B(1-807)-GFP-FRB, and KIF17(1-547)-GFP-FRB have been published previously[4]. pTagRFP-Tubulin vector was purchased from Evrogene and mCherry-α-tubulin is a gift of Dr. R.Tsien[5].

Expression and purification of recombinant proteins in E.coli

Recombinant bacterially expressed bio-VHH_{GFP} was obtained by using IMPACT Intein purification system. Induction, expression and purification of fusion proteins were performed according to the manufacturer's instructions (New England Biolabs). Briefly, E.coli strain BL21(DE3) was transformed with the plasmid encoding 6xHis-SUMO-BirA and bio-VHH_{GFP}-Intein-CBD and grown at 37°C overnight in the presence of 100 µg/mL of ampicillin and kanamycin. For production of biotinylated proteins, overnight bacterial culture was diluted (1:100) in fresh LB media containing 0.1% D-glucose, 50 µM D-Biotin, and ampicillin and grown till OD600 reached 0.6-0.8. Expression of recombinant proteins was induced with 0.5 mM IPTG at 37°C for FKBP and at 25°C for VHH_{GFP} and VHH_{GFP}(2x). After 3 hours cells were lysed in cold Column buffer (20 mM Na-HEPES, pH 8.5, 250 mM NaCl) containing 0.1 - 0.5% Triton X-100 and Protease inhibitor cocktail (Roche), sonicated and purified on Chitin beads according to the manufactures protocol. Beads bound proteins were cleaved at 4°C overnight with 10-20 mM of DTT in Column buffer and then immediately dialyzed into PBS, aliquoted and stored at -80°C. Cleavage efficiency and purity were estimated by SDS-PAGE

and coomassie blue staining. Biotinylation of purified proteins was confirmed by western blot with Streptavidin coupled to HRP (1:20.000; Pierce) (see Supplementary Fig 1d). 6xHis-SUMO-GFP was induced for 3 hours at 37°C and purified with Probond resin (Invitrogen) according to a standard protocol. Purified protein was eluted from the beads by Imidazole gradient (150-300 mM), dialyzed into PBS, aliquoted and stored at -80°C.

Cell culture, transfections and immunocytochemistry

COS-7 were cultured at 37°C in DMEM/Ham's F10 (50/50%) medium supplemented with 10% FCS and 1% penicillin/streptomycin. 1-3 days before transfection, cells were plated on 19 or 24 mm diameter glass coverslips. Cells were transfected with Fugene6 transfection reagent (Roche) according to the manufacturers protocol and grown for 16-24 hours. Fixation was done with 4% PFA for 15 min at RT, washed with PBS and permeabilized with 0.25% Triton X-100 in PBS for 10 min. After extensive washing in PBS coverslips were mounted on microscopic slides. Primary monoclonal mouse anti-acetylated tubulin antibody (1:400; Sigma#T7451, clone 6-11B-1) and rat monoclonal anti-tyr-tubulin antibody (1:500; Abcam#ab6160 [YL1/2]) were added and incubated overnight at 4°C. Next, coverslips were washed in PBS and incubated with secondary anti-mouse Alexa Fluor 568 and anti-rat Alexa Fluor 647 antibody (Molecular Probes) diluted in blocking buffer (1:500) for 1 hour at RT. After washing in PBS coverslips were mounted on microscopic slides with Moviol.

Electroporation of COS-7 cells and functionalization of QDs

For electroporation of plated adherent COS-7 cells (all values are given for one 24 or 25 mm coverslip), 2 µl of Qdot 625 streptavidin conjugate (1 µM; A10196, Molecular Probes, Life sciences) and 20-25 µl of purified bio-VHH_{GFP} (0.7-0.8 µg/µl) were diluted in PBS to a final volume of 200 µl. Reaction was incubated for 1 hour at room temperature and then at 4°C overnight. Cells were electroporated with the Nepa21 Electroporation system (Nepagene) using CUY900-13-3-5 cell-culture-plate electrode with 5 mm distance between electrodes. If using different electroporation systems or electrodes shapes, it is possible to recalculate provided values by keeping voltage per cm ratio in parameters the same. Electroporation was performed in 6-well plate containing 1.8 ml of warm Ringer's solution (10 mM Hepes, 155 mM NaCl, 1 mM $CaCl_2$, 1 mM $MgCl_2$, 2 mM NaH_2PO_4, 10 mM glucose, pH 7.2) and 200 µl of electroporation mix per well. In different electroporation systems the volume of Ringer's solution can be decreased to increase QDs concentration and consequently, electroporation efficiency. In our system 2 ml constitutes the minimal volume required for electrodes to be

immersed in solution. Parameters for electroporation (Voltage, Interval, Decay, Number and Pulse Length) were optimized from standard settings to achieve optimal efficiency and provided in Table S1. We found that the number of incorporated QDs per cell mainly is proportional to the pouring pulse voltage, length (duration) and repetition. At the same time increase in those values leads to the overall decreased cell survival. Each coverslip was electroporated with fresh solution of QDs. Electroporation program was applied two times, rotating electrod 90 degrees for the second time. Cells were then washed three times with Ringer's solution to remove QDs from solution and either mounted with growth medium in imaging ring chamber for immediate live imaging experiments or returned back to the growth medium and fixed at different time points. Detailed protocol is available online[6].

Live cell imaging of targeted QD (Fig. 2 b-g)

Live imaging of COS-7 cells with QD targeted to proteins of interest (Fig. 2 b-g) was performed on a Nikon Eclipse TE2000E (Nikon) equipped with an incubation chamber (Tokai Hit; INUG2-ZILCS-H2) mounted on a motorized stage (Prior). Coverslips (24 mm) were mounted in metal rings, immersed in 0.8 ml Ringer's solution and maintained at 37ºC. For imaging of kinesin-driven transport TIRFM was performed using a 100x objective (Apo TIRF, NA=1.49, Nikon), and EMCDD camera (Evolve512, Photometrics). TIRF angle was adjusted manually so that the sample illumination was either wide-field or highly oblique. A 488 nm Argon laser line was used for excitation of both GFP and QDs. A DualView DV2 (Photometrics) was used to image GFP and QDs side-by-side. Movies were recorded as stream acquisitions with 50 ms, 100 ms or 200 ms exposure.

Rapid frame rate imaging of QD in living cells (Fig. 1 c-g)

Electroporation of streptavidin-conjugated QDs in COS-7 cells was performed as described above. The actin cytoskeleton disrupting agent Latrunculin A was solubilized in DMSO (10 mM stock solution) and applied to cells in Ringer's solution (or DMEM/F10) to the final concentration of 10 μM for 40 minutes. Live-cell acquisitions were performed on Nikon Eclipse Ti-E microscope with a perfect focus system (Nikon) equipped with Nikon CFI Apo TIRF 100×, 1.49 N.A. oil objective (Nikon) and controlled with μManager software[7]. Wide-field or oblique laser illumination was achieved using a 100mW 561 nm DPSS laser (Cobolt Jive) and a set of mirrors. QD fluorescence was collected through a ET595/50 emission filter (Chroma) with a water-cooled Neo sCMOS camera (Andor) with 512x512 pixel ROI. The final image pixel size was equal to 65 nm. Stream movies were recorded with 2.4 ms exposure for

2000 frames. For each condition (before and after Latrunculin A addition) we performed 2 independent experiments, imaged 3-5 cells per condition and recorded 3 movies per cell.

Laser confocal imaging of fixed COS-7 cells (Fig.3e, S3b)

Confocal images from transfected, fixed and immunostained COS-7 (Fig. 3e and Supplementary Fig. 3b) were acquired on Leica TCS SP5 scanning system equipped with Diode (405nm), Argon (458, 476, 488, 496, 514 nm laser lines), Diode Pumped Solid State (DPSS, 561nm) and HeNe (633nm) lasers and acousto-optic tunable filters (AOTF) for selection and intensity adaptation of laser lines using LAS AF (Leica Application Suite Advanced Fluorescence) imaging software. Cells were imaged as z-stacks using a 63x 1.4NA oil immersion objective and confocal zoom factor 2-6 and displayed as projections of maximum intensity.

Confocal spinning disk imaging (Fig. 1a-b, 3g-f, 4a-b)

Spinning disk microscopy was performed on an inverted microscope Nikon Eclipse Ti-E (Nikon) with the perfect focus system (PFS) (Nikon), equipped with Plan Apo VC 100x N.A.1.40 and Plan Apo 60x N.A.1.40 oil objectives (Nikon), CSU-X1-A1 Spinning Disc (Yokogawa) and Photometrics Evolve 512 EMCCD camera (Roper Scientific) and controlled with the MetaMorph 7.7 software (Molecular Devices). Imaging was performed using an Evolve 512 camera with intermediate lens 2.0X (Edmund Optics) at a magnification of 0.065 (100x) and 0.011 (60x) µm/pixel. The microscope was equipped with a custom-ordered illuminator (Nikon, MEY10021) modified by Roper Scientific France/PICT-IBiSA, Institut Curie. For fluorescence excitation a 491 nm 100 mW Calypso (Cobolt) and a 561 nm 100 mW Jive (Cobolt) laser were used. The spinning disk was equipped with 405-491-561 triple band mirror and GFP and mCherry emission filters (Chroma).

For imaging of QDs and actin cytoskeleton COS-7 cells (Fig.1 a,b) were electroporated with QDs as described above and after 30 minutes fixed with 4% formaldehyde in PBS solution. Actin was stained with Alexa Fluor 488 Phalloidin (A12379, Molecular Probes) according to manufacturer’s protocol. Z-stacks were acquired in each channel (GFP and mCherry) with 60x objective and 300 nm spacing between planes (25-30 planes in total per stack). Stacks were deconvolved using the Huygens Professional package (Scientific Volume Imaging) and 3D Gaussian Blur of 1 pixel was applied using ImageJ.

We used the same setup for live imaging of microtubules only (for iMSD analysis, Fig. 4a,b) or simultaneous kinesin and microtubules (Fig. 3f,g). COS-7 cells were either transfected with and

mCherry-α-tubulin only or co-transfected with TagRFP-Tubulin and KIF5B-GFP-FRB or KIF21B-GFP-FRB for 24 hours. Imaging was performed in Ringer's solution at 37°C and 5% CO2 (by using stage top incubator INUBG2E-ZILCS (Tokai Hit)). Two fluorescent channels (GFP and mCherry) were acquired consecutively with 1 s interval between frames and 100-200 ms exposure for 300 frames. Imaging was performed at one z-plane. For simultaneous kinesin and microtubule imaging movies were corrected for bleaching and background subtracted (using rolling ball of 10 pixel radius). For actin-disrupting treatments stock solutions of latrunculin A, jasplakinolide and blebbistain were diluted in cell's culture medium to the final concentration of 10 μM, 10 μM and 50 μM respectively and applied to cells for 40 minutes before imaging.

Stoichiometric quantification of GFP binding to QD-VHH$_{GFP}$

Glass coverslips were sonicated in an ultrasonic bath (sonicator Soniprep 150) for 10 min in ultrapure water and then air-dried. Three flow chambers with an approximate volume of 5 μL each were made with four stripes of double-sided tape between a cleaned 22x22 mm coverslip and the microscope slide. 5 μl of Electroporation mix containing QDs, bio-VHH$_{GFP}$ (or bio-VHH$_{GFP}$(2x)) and PBS (same as described above) and 1 μl of purified 6xHis-SUMO-GFP (diluted to 100 ng/μl) were mixed in 94 μl of PBS (Mix1 and Mix2, respectively) and incubated for 30 min at RT. Two chambers on the coverslip were incubated with poly-L-lysine (PLL; 0.2 mg/mL) for 3 min. After washing with 20 μl of PBS, the surface was blocked with κ-casein (1 mg/mL), further washed with PBS and then incubated with Mix1 or Mix2 for 3 min. The third flow chamber was used for preparation of single immobilized GFP molecules. First, the surface was coated with 0.2 mg/mL biotinylated poly(L-lysine)-[g]-poly(ethylene glycol) (PLL-PEG-biotin, 0.2 mg/mL; Susos), washed with 20 μl of PBS, and incubated for 3 min with 1 mg/mL NeutrAvidin. bio-VHH$_{GFP}$ (10 ng/μl) was immobilized via NeutrAvidin-biotin interaction. Unspecific surface protein binding was blocked by incubation with κ-casein (1 mg/ml) for 3 min and further washed with PBS. Purified 6xHis-SUMO-GFP (0.1 ng/μl) was added and incubated for 3 min at RT. Unbound GFP molecules and QDs were removed by extensive wash with PBS. All steps were performed at the RT. Chambers were subsequently sealed with vacuum grease and immediately proceeded to imaging.

These samples were imaged on an inverted microscope (Nikon Eclipse Ti-E; Nikon) with perfect focus system (Nikon), equipped with Nikon CFI Apo TIRF 100×, 1.49 N.A. oil objective (Nikon), Photometrics Evolve 512 EMCCD (Roper Scientific), and controlled with MetaMorph 7.7.5 software (Molecular Devices). The microscope was equipped with a TIRF-E

motorized TIRF illuminator. For excitation of GFP we used a 491 nm Calypso (Cobolt) laser and a 561 nm Jive (Cobolt) laser was used for QDs. We used ET-GFP and ET-mCherry filter sets (Chroma) for consecutive imaging of GFP and QDs, respectively. The 16-bit images acquired using an Evolve 512 camera placed behind an intermediate 2.5× lens (Nikon C mount adapter 2.5×). The final pixel size was 0.065 μm. Stream movies with 100 ms exposure were acquired in the GFP channel for 1000 frames. Total bleaching of GFP fluorescence was observed in the end of acquisition. 100 frames with exposure of 100 ms were acquired in QDs channel to register QDs positions in spite of their blinking. All acquisition parameters (laser intensities and beam angle, exposure, camera EM gain, etc) were kept constant during successive imaging of chambers.

Analysis routines were implemented in ImageJ as macros or Java plugins. The location of QDs was determined from maximum intensity projections of stream QD movies. Only the first (unbleached) frames of GFP channel recordings were used for intensity quantifications. A GFP cluster was considered to be colocalized with a QD if the distance between maximum intensity pixels of the fluorescent spots was less than 1.5 pixels. The measurements of integrated fluorescence intensity without background (Fig. S1f) were performed similar to method described previously[8]. We counted raw integrated intensity I_R of 13 x 13 pixel region of area S_R that was centered on the maximum intensity pixel of a fluorescent spot. The raw integrated intensity of background I_B was equal to integrated counts of 14 x 14 pixel region of area S_B minus I_R. The final integrated fluorescence intensity (without background) I_F was equal to:

$$I_F = I_R - I_B \frac{S_R}{S_B}$$

The characteristic distribution of intensities from one experiment is presented at Fig. S1f. We pooled together results of three independent experiments to calculate the average number of GFP molecules bound to QDs reported in the main text. The values of integrated intensity of the GFP clusters colocalized with QDs-VHH_{GFP} and QDs-VHH_{GFP}(2x) from each experiment was normalized by the average integrated intensity of corresponding single GFP. We calculated mean values and standard deviation for resulting pooled GFP molecules counts for QDs-VHH_{GFP} (N=2941) and QDs-VHH_{GFP}(2x) (N=5591). The bleaching intensity traces (Fig.S1g,h) were filtered using Chung-Kennedy nonlinear edge-preserving filter with parameter values K = 1, M = 10, p = 4[9].

Particle detection and trajectory analysis

Image processing routines were automated using ImageJ/FIJI macros or custom build plugins.

MSD calculation, curve fitting and all other statistical and numerical data analysis were performed in Matlab (MATLAB R2011b; MathWorks) and GraphPad Prism (ver.5.02, GraphPad Software).

Particle detection and tracking. To track and characterize individual quantum dots movements we used TrackMate plugin (v.2.5.0) for FIJI with subpixel LoG detector and "Simple LAP tracker" option[10]. Resulting trajectories were exported to MTrackJ ImageJ plugin[11] for manual inspection and correction. To get localization precision, each detected spot was further fitted with 2D Gaussian with initial parameters corresponding to the microscopes point spread function as described earlier using ImageJ Dom_Utrecht plugin v.0.9.2 [12]. Only tracks longer than 12 (for diffusion) and 50 (for QD-kinesin trajectories) frames were selected for the further analysis.

Diffusion trajectory analysis. MSD and velocity autocorrelation curves together with diffusion coefficient calculations were performed using "msdanalyzer" Matlab class[13]. Ensemble diffusion coefficients were measured as a slope of the affine regression line fitted to the first 25% of weighted averaged MSD curves and divided by four (assuming two dimensional motion).

Motors runs analysis. Detection of processive runs in trajectories was performed using two algorithms. The first one, called "strict directional filtering", is calculating the cosine between two consecutive velocity vectors for a given trajectory and finds segments where its value is above defined threshold. In this segment, the particle is assumed to move directionally. Directional autocorrelation is used as a local directional persistence measure in multiple applications[13]. Each trajectory represents a set of particle coordinates $\vec{r}_i$, where index *i* denotes the frame number. Corresponding velocity vectors were defined as $\vec{v}_i = (\vec{r}_{i+1} - \vec{r}_i)/\tau$, where τ is the time between frames. The value of cosine was calculated as:

$$\cos\alpha_i = \frac{(\vec{v}_i, \vec{v}_{i-1})}{|\vec{v}_i||\vec{v}_{i-1}|}$$

To find runs we used the lower threshold value of 0.6, corresponding to an approximately 100° cone facing forward. Only runs longer than 0.5 seconds were taken into account. To address the efficiency of the algorithm we tested it on a dataset containing 500 artificial trajectories of 1000 frames each (20 frames per second). Each track represented diffusive motion with coefficient D=0.2 $\mu m^2/s$ interrupted by four randomly located continuous runs with exponential distribution of durations. During processive runs the speed was constant and the change of the angle between two consecutive velocity vectors was sampled from a Gaussian distribution with

standard deviation of 15°. The efficiency of detection did not depend on the speed of runs (data not shown), but declined as the duration of runs became shorter, as shown on Fig. S2e. At the average duration of run equal to one second (comparable to characteristic experimental kinesin run length) the algorithm detected correctly 94% of frames containing processive movements with only 6% of false positives. For the velocity and length analysis (Fig.2i-k and Supplementary Table 3) we used this algorithm with threshold value of 0.6 and minimal number of frames in one run equal to 10.

The second algorithm, referred to as "multi-scale filtering", was developed to detect kinesin runs in the presence of microtubules displacements. In this case, processive runs maintain general direction of movement but can be locally disrupted by abrupt random movements. To overcome the strict local criteria of the first algorithm we used "non-local" directionality measure as:

$$\cos\alpha_{i,k} = \frac{(\vec{r}_{i+k} - \vec{r}_i, \vec{r}_i - \vec{r}_{i-k})}{|\vec{r}_{i+k} - \vec{r}_i| \cdot |\vec{r}_i - \vec{r}_{i-k}|}$$

where positive integer k defines a window (time scale) of directionality. To find this kind of "disrupted" runs we used its average value over multiple time scales:

$$< \cos\alpha_i >_K = \frac{1}{K}\sum_{k=1}^{K} \cos\alpha_{i,k}$$

Using this characteristic with K=9, we initially located those segments of trajectory where its value was above -0.1 and among them we picked only those longer than one second, where the average value of $< \cos\alpha_i >_K$ along segment was above 0.6. To test the efficiency of the algorithm we used similar artificial trajectory dataset, but additional random direction displacement was added during processive runs. The magnitude of this displacement was exponentially distributed with the average value equal to the half the processive speed movement per frame. The results presented on Fig. S2f show that "multi-scale filtering" algorithm robustly detects periods of directed motion in trajectories over the wide range of runs durations. It performs better on longer durations, since the short ones are often filtered out due to the time-scale of the filtering window. We used the same algorithm's parameters to analyze QD-kinesin trajectories.

Spline fitting and curvature calculations. Segments of directional runs were fitted with non-periodic cubic B-splines using "B-splines" Matlab package by Levente Hunyadi (http://www.mathworks.com/matlabcentral/fileexchange/27374-b-splines). In short, first the length of the run L was estimated as a total sum of displacements. The degree of approximation

was defined by the average distance between spline's control points denoted l. The total number of control points was defines as n=[L/l]+1 and the number of knots as p = n + 6. The knot vector **t** points were equally spaced:

$$t_1 = t_2 = t_3 = t_4 = 0, t_i = \frac{1}{n-1}, t_{p-3} = t_{p-2} = t_{p-1} = t_p = 1$$

and corresponding control point positions were determined by the iterative minimization of the sum of distances between trajectory points and the B-spline. Decomposition to the longitudinal and transverse components was performed using distance2curve (http://www.mathworks.com/matlabcentral/fileexchange/34869-distance2curve) Matlab function by John D'Errico. To calculate the curvature, fitted B-spline curve was evenly split along its arc length into segments of 50 nm. Each three consecutive points were fitted with the circle and inverse of its radius was used as the curvature measure.

All source code of Matlab routines for the motor runs detection, artificial trajectories generation, B-spline fitting and curvature calculations is available online[14].

Measuring Stokes radius of QDs (Fig. S1a)

QDs where diluted to a final concentration of 1 nM in various aqueous solutions of glycerol. We used dilutions of 0, 10, 20, 30, 40, 50, 60 and 70% volume glycerol concentrations in water. A final volume of 100 µm was placed on 24x50 mm coverslip and imaged on the same setup and with the same parameters as described for Fig. 1c-f (see "Rapid frame rate imaging" section) at 20°C. 5 movies of 1500 frames each were collected for each concentration. QD trajectories for each condition were built as described in the previous section. Diffusion coefficients were derived from linear fitting of averaged MSD curves (200-600 trajectories per each concentration). The dynamic viscosity coefficient η for each dilution was measured at 20°C using a rotational rheometer (MCR 300, Anton Paar GmbH, Graz, Austria). Within measurement error, the obtained values were consistent with previously reported values[15].

Analysis of QD blinking (Fig. S1c)

"In vitro" measurements of QDs blinking were performed by first diluting stock QD solution to 10 nM in PBS and running it through a flow chamber of the same design as in the "*Stoichiometric quantification*" section. As a result QDs were non-specifically immobilized on the coverslip. The flow chamber was further filled with imaging medium (PBS or 10mM MEA in PBS), sealed with vacuum grease and imaged on the same setup and with the same parameters as described for Fig. 1c-f (see "Rapid frame rate imaging" section). Three movies of

1000 frames were acquired per condition. Durations of "on"-time segments were calculated from tracks as periods of continuous QDs emission. The cumulative distribution function of these durations is shown on Fig. S1c. For the "inside cell" condition we used the slow diffusing subpopulation of QDs also used in the dataset presented in Fig.1d-f, analyzed in the same manner.

iMSD kymograph analysis (Fig.4b-e)

Kymographs of microtubule transverse displacements (Fig.4b) were built using KymoResliceWide v.0.4 ImageJ plugin[16]. Kymograph images were loaded to Matlab and 1D spatiotemporal image correlation function was calculated according to [17]:

$$G(\chi,\tau) = \frac{\langle I(x,t)\cdot I(x+\chi,t+\tau)\rangle}{\langle I(x,t)\rangle^2} - 1$$

For each time point, we subtracted the G value at $\tau = T = 50$ s to exclude the immobile component (background, diffuse fraction of tubulin) and fitted the resulting curve with a Gaussian function:

$$G(\chi,\tau) - G(\chi,T) = A\exp\left(-\frac{\chi^2}{\sigma_r^2(\tau)}\right)$$

The value of $\sigma_r^2(\tau) - \sigma_0^2$ is plotted as iMSD (image mean square displacement) on Fig.4 and it was fitted with a simplified formula for MSD versus time lag τ for confined motion[17, 18]:

$$\mathrm{iMSD}(\tau) = \frac{L}{6}\left(1 - \exp\left(-\frac{12D}{L^2}\tau\right)\right),$$

where L characterizes the size of confinement and D is the diffusion coefficient of motion on length scales far below L. The iMSD for each kymograph was fitted separately and the summary of L and D values for each condition is presented on Fig.4d-e. Parameters of fits which provided the value of confinement size L larger than approximate cell size of 20 µm were excluded from statistical comparison.

SUPPLEMENTARY NOTE 1

Actin pore size estimation

According to Eq.9 in reference[19], for diffusion of a tracer with radius R through a network of filaments of radius r (when R size is compatible with r) , the inaccessible volume fraction is:

$$\phi_{\mathrm{eff}} = 1-(1-\phi)^{(1+R/r)^2}$$

where ϕ is the volume fraction of overlapping filaments and can be approximated as $\phi = \frac{3\pi r^2}{L^2}$ for a regular cubic filament grid with cell's edge length L. Using the values for $D_{\mathrm{eff}}/D_{\mathrm{o}}$ = $D_{\mathrm{slow}}/D_{\mathrm{fast}}$, a QD radius of R=15 nm, actin filament radius r=4 nm, we numerically solved the system of equations Eq.9-10 in [19] to estimate the pore size being equal to L=36 nm.

SUPPLEMENTARY REFERENCES

1. Caussinus E, Kanca O, Affolter M. Fluorescent fusion protein knockout mediated by anti-GFP nanobody. *Nat Struct Mol Biol* **19**, 117-121 (2012).

2. Lansbergen G*, et al.* CLASPs attach microtubule plus ends to the cell cortex through a complex with LL5beta. *Dev Cell* **11**, 21-32 (2006).

3. Okamoto K, Nagai T, Miyawaki A, Hayashi Y. Rapid and persistent modulation of actin dynamics regulates postsynaptic reorganization underlying bidirectional plasticity. *Nat Neurosci* **7**, 1104-1112 (2004).

4. Kapitein LC*, et al.* Mixed microtubules steer dynein-driven cargo transport into dendrites. *Curr Biol* **20**, 290-299 (2010).

5. Shaner NC, Campbell RE, Steinbach PA, Giepmans BN, Palmer AE, Tsien RY. Improved monomeric red, orange and yellow fluorescent proteins derived from Discosoma sp. red fluorescent protein. *Nat Biotechnol* **22**, 1567-1572 (2004).

6. Katrukha EA*, et al.* Electroporation of COS-7 cells and functionalization of QD, http://dx.doi.org/10.17504/protocols.io.g2mbyc6 (2017).

7. Edelstein AD, Tsuchida MA, Amodaj N, Pinkard H, Vale RD, Stuurman N. Advanced methods of microscope control using muManager software. *J Biol Methods* **1**, (2014).

8. Hoffman DB, Pearson CG, Yen TJ, Howell BJ, Salmon ED. Microtubule-dependent changes in assembly of microtubule motor proteins and mitotic spindle checkpoint proteins at PtK1 kinetochores. *Mol Biol Cell* **12**, 1995-2009 (2001).

9. Chung SH, Kennedy RA. Forward-backward non-linear filtering technique for extracting small biological signals from noise. *J Neurosci Methods* **40**, 71-86 (1991).

10. Schindelin J*, et al.* Fiji: an open-source platform for biological-image analysis. *Nat Methods* **9**, 676-682 (2012).

11. Meijering E, Dzyubachyk O, Smal I. Methods for cell and particle tracking. *Methods Enzymol* **504**, 183-200 (2012).

12. Doodhi H, Katrukha EA, Kapitein LC, Akhmanova A. Mechanical and geometrical constraints control kinesin-based microtubule guidance. *Curr Biol* **24**, 322-328 (2014).

13. Tarantino N*, et al.* TNF and IL-1 exhibit distinct ubiquitin requirements for inducing NEMO-IKK supramolecular structures. *J Cell Biol* **204**, 231-245 (2014).

14. Katrukha EA*, et al.* Probing cytoskeletal modulation of passive and active intracellular dynamics using nanobody-functionalized quantum dots. Datasets, code and figures, https://dx.doi.org/10.6084/m9.figshare.c.3672244.v1 (2017).

15. Segur JB, Oberstar HE. Viscosity of Glycerol and Its Aqueous Solutions. *Industrial and Engineering Chemistry* **43**, 2117-2120 (1951).

16. Katrukha EA. ImageJ plugin making kymographs of maximum or average intensity with wide lines, polylines, curves, https://github.com/ekatrukha/KymoResliceWide (2017).

17. Di Rienzo C, Gratton E, Beltram F, Cardarelli F. Fast spatiotemporal correlation spectroscopy to determine protein lateral diffusion laws in live cell membranes. *Proc Natl Acad Sci U S A* **110**, 12307-12312 (2013).

18. Kusumi A, Sako Y, Yamamoto M. Confined lateral diffusion of membrane receptors as studied by single particle tracking (nanovid microscopy). Effects of calcium-induced differentiation in cultured epithelial cells. *Biophys J* **65**, 2021-2040 (1993).

19. Novak IL, Kraikivski P, Slepchenko BM. Diffusion in Cytoplasm: Effects of Excluded Volume Due to Internal Membranes and Cytoskeletal Structures. *Biophys J* **97**, 758-767 (2009).
